# An Aim at Looking Forward More Wisely

**Published:** 1889-10

**Authors:** E. D. Babbitt

**Affiliations:** N. Y. College of Magnetics, 50 Union Square


### AN AIM AT LOOKING FORWARD MORE WISELY.

BY E. D. BABBITT, M.D.

An intelligent writer, by the name of Mr. S. H. Preston, has an article in the September number of HALL'S JOURNAL entitled "Looking Forward." The writer brings up an array of figures to show that the world will in a few centuries become vastly overcrowded, so that the people must inevitably starve to death and all things come to a common ruin. My aim shall be to show that this horrible result is entirely imaginary and founded on false reasoning.

The writer says that population increases in geometrical progression, doubling every twenty-five years, and shows that this rate of increase will place over 35,000 times the present population of the globe in our country alone in fifteen centuries. Of course such a population could scarcely find standing room, much less room to support themselves by agriculture or manufactures. Supposing the population to double only three times in a century, he has correctly figured out a population of more than twenty-six billions, which is about twenty-two times the present population of the globe. Of course such a number would eat one another up, and all that the land could raise besides. If this is true, and if our destruction shall be accomplished in two or three centuries, then there is poor encouragement for building up any grand institutions or working for humanity, for the whole constitution of things is a miserable failure and the world should be blown up as soon as possible before this fearful starvation scene commences.

But let us see how this theory harmonizes with the facts of the world's history. The truth is that no such rate of progress, or the tenth part of it, rules among nations as a whole. With such a rate of progress the world would be crowded to death in about two centuries, and the great continents and islands would have been left to silence thousands of years ago. Henry George has brought up quite an array of facts to show that population as a whole does not increase at all. In this Mr. George is no doubt mistaken. To be sure, there are many nations and cities in their early history that may increase rapidly for a while, but not geometrically, except for a limited time. When overcrowding takes place the increase is checked. Malthusianism is one of the most absurd pieces of sophistry that the world has ever seen. The Rev. Mr. Malthus wrote nearly a century ago, and according to his theory of populations doubling every twenty-five years the world should be nearly destroyed by this time. His theory has been shrewdly knocked to pieces by an application to the family and descendants of Confucius. On the supposition that population doubles every twenty-five years, these descendants of therevered Chinese sage should have amounted to 859,559,193,106,709,670,198,710,528 souls in 2,150 years after the death of Confucius, a number large enough to people millions of solar systems! So much for theory. What are the facts? History says that in the reign of Kanghi, 2,150 years after the death of Confucius, his male descendants were 11,000, which would show that 22,000 persons would cover the whole number. Theory thus overleaps the truth of things by being nearly forty septillion times too much.

These sensational Malthusian theories were an outgrowth from Adam Smith's teachings, and were swallowed whole by such eminent writers as Mill, Buckle, Carlyle, and a host of others.

But our writer goes on to show the fearful dangers of "over-population," and declares that "the number of workers already far exceeds the demand for work," there being a million idlers. But here again is a mistake. The densest population in the United States is in Massachusetts, which according to the census of 1880 was 229 to a square mile. In this very State, however, which is so crowded, the average wealth to each individual has always heretofore been greater than that of any other State and may be so still, although I have not the statistics of the last census by me. It is high time that the world should understand the fact that *the basis of wages is labor*, *not capital*, for which reason there will be no danger of having too many laborers for a thousand years to come, *if we can only bring about a righteous division and distribution of the results of labor*. Our present system allows the strong, the cunning, and the rapacious to devour those who are weak or who are engaged in some nobler work than mere money making, and hence cannot cope with the tricks of trade. Let us look at causes, not forever skim the surface of things. With our vast new machinery, which is more and more crowding out human labor, the people must inevitably be driven to ruin unless they organize and employ themselves, in other words, *co-operate.* It can be demonstrated that laborers do not get one-fifth of what they earn even in America. Two or three years since the Labor Bureau showed that each laborer earned, directly or indirectly, $10 a day, but received only $1.15. Keep the laborer down, house him in a kind of pig-sty without means for ennoblement or happiness, and it will make him lose self-respect and drive him into drinking and animalism. In Europe thousands of co-operative societies have sprung into being and are working with marvelous efficiency for human good. Mr. A. K. Owen's remarkable work called "Integral Co-operation" opens up the true pathway of human redemption, and the Credit Foncier Company, founded on its principles, has its colony working harmoniously on the beautiful Topolobampa Bay and vicinity in Western Mexico, in spite of the gigantic falsehoods of a perverted press. The Nationalist party, founded on that grand work of Mr. Bellamy called "Looking Backward," has sprung swiftly into being in various parts of the United States and proclaims a gospel of glad tidings to the suffering people. Is it right to call such movements utopian and visionary? No, from the fact that these same principles have been tested by the Familistère of Guise, France, for twenty-seven years, with a harmonizing, happifying and beautifying effect upon all its members, now nearly 2,000 in number, which is simply marvelous. Outside of their sacred precincts the laboring people are apt to live in hovels, sometimes in windowless rooms, with squalid surroundings and ragged, uneducated children, while inside the people live in their palace, of which they are rapidly becoming owners, have park like surroundings, a theater, a wonderful system of schools and nurseries, and baths and festivals, and those co operative arrangements which lift so many burdens of life. Among all these happy people there never has been a pauper or a criminal, and the drunkenness which so often marks the discouraged laborer in the present perverted system of life, has disappeared. Let the croakers then, who are always crying out "visionary," "unpractical," hold their peace in the light of such facts, and let our writers cease to advocate the impending ruin that is coming upon us, becoming enkindled with the grander facts of the living present, which shall prevent all ruinous tendencies and lift man to his true and glorious destiny.

N. Y. College of Magnetics, 50 Union Square

